# Choline elevation in amygdala region at recovery indicates longer survival without depressive episode: a magnetic resonance spectroscopy study

**DOI:** 10.1007/s00213-019-05303-2

**Published:** 2019-09-04

**Authors:** Neven Henigsberg, Aleksandar Savić, Marko Radoš, Milan Radoš, Helena Šarac, Ana Šečić, Maja Bajs Janović, Tamara Foro, David Ozretić, Viktorija Erdeljić Turk, Pero Hrabač, Petra Kalember

**Affiliations:** 1 Croatian Institute for Brain Research, School of Medicine, University of Zagreb, Šalata 12, 10000 Zagreb, Croatia; 2 University Psychiatric Hospital Vrapče, Zagreb, Croatia; 3 Croatian Institute for Brain Research, Centre of Excellence for Basic, Clinical and Translational Neuroscience, University of Zagreb School of Medicine, Zagreb, Croatia; 4 School of Medicine, University of Zagreb, Zagreb, Croatia; 5 University Hospital Centre Zagreb, Zagreb, Croatia; 6 University Hospital Centre ‘Sestre Milosrdnice’, Zagreb, Croatia; 7 “Andrija Štampar” School of Public Health, School of Medicine University of Zagreb, Zagreb, Croatia; 8 Polyclinic Neuron, Zagreb, Croatia

**Keywords:** Depression, Magnetic resonance spectroscopy, Recurrence, Antidepressants, Therapy, Recovery, NAA, Cho, Glx

## Abstract

**Rationale:**

Depression, with variable longitudinal patterns, recurs in one third of patients. We lack useful predictors of its course/outcome, and proton magnetic resonance spectroscopy (1H-MRS) of brain metabolites is an underused research modality in finding outcome correlates.

**Objectives:**

To determine if brain metabolite levels/changes in the amygdala region observed early in the recovery phase indicate depression recurrence risk in patients receiving maintenance therapy.

**Methods:**

Forty-eight patients on stable-dose antidepressant (AD) maintenance therapy were analyzed from recovery onset until (i) recurrence of depression or (ii) start of AD discontinuation. Two 1H-MRS scans (6 months apart) were performed with a focus on amygdala at the beginning of recovery. N-acetylaspartate (NAA), choline-containing metabolites (Cho), and Glx (glutamine/glutamate and GABA) were evaluated with regard to time without recurrence, and risks were assessed by Cox proportional hazard modeling.

**Results:**

Twenty patients had depression recurrence, and 23 patients reached AD discontinuation. General linear model repeated measures analysis displayed three-way interaction of measurement time, metabolite level, and recurrence on maintenance therapy, in a multivariate test, Wilks’ lambda = 0.857, *F*(2,40) = 3.348, *p* = 0.045. Cho levels at the beginning of recovery and subsequent changes convey the highest risk for earlier recurrence. Patients experiencing higher amygdala Cho after recovery are at a significantly lower risk for depression recurrence (hazard ratio = 0.32; 95% confidence interval 0.13–0.77).

**Conclusion:**

Cho levels/changes in the amygdala early in the recovery phase correlate with clinical outcome. In the absence of major NAA fluctuations, changes in Cho and Glx may suggest a shift towards reduction in (previously increased) glutamatergic neurotransmission. Investigation of a larger sample with greater sampling frequency is needed to confirm the possible predictive role of metabolite changes in the amygdala region early in the recovery phase.

## Introduction

Following the initial episode, depression recurs in approximately 35% of patients. Remission is considered to begin after 3 consecutive weeks of minimal symptom status. Recovery is reached if stable symptom status continues for at least 4 months. Recurrence is when another depressive episode emerges during the recovery phase (Rush et al. 2006). In general, recurrence risk is increased by the number of past episodes and decreased by a longer time span after the previous episode, although there is considerable intra-individual variation (Solomon et al. 2000). Other recognized risks include previous history of dysthymic disorder, early onset, residual depressive symptoms, and family history of recurrent depression (APA 2010). Patients who experienced three or more prior depressive episodes should continue maintenance therapy in the continuation phase at the full therapeutic dose used to achieve remission; otherwise, the risk of recurrence approaches 100%.

Proton magnetic resonance spectroscopy (1H-MRS) evaluates brain metabolites in vivo, measuring their quantities against standard metabolic curves. 1H-MRS is most widely used modality because of its high sensitivity, high signal-to-noise ratio, and the use of standard radiofrequency coils (van der Graaf 2010). Neurochemicals identified by 1H-MRS include N-acetyl aspartate (NAA), creatine (Cr), choline-containing metabolites (Cho), Glx peak-containing glutamine/glutamate and γ-aminobutyric acid (GABA), myoinositol, glycine, glucose, lipids, lactate, alanine, acetate, succinate, and taurine.

NAA is considered a putative marker of neuronal integrity (Stanley 2002) and functionality (Sager et al. 2001; Tsai and Coyle 1995), as well as an MRS marker for neuronal health, viability, and number (Moffett et al. 2007). Several studies reported altered NAA levels following antidepressant (AD) treatment in various brain regions (Caverzasi et al. 2012). Cho is considered a potential marker of membrane phospholipid metabolism and membrane turnover (Frahm et al. 1989; Petroff 1988; Stanley et al. 2000), and increased Cho levels in depression therapy responders do not seem to be medication specific (Bernier et al. 2009; Luborzewski et al. 2007; Zhang et al. 2015) and appears to be resistant to some comorbidities (Henigsberg et al. 2011).

The finding of decreased Glx in depressed patients is frequently described, but it is inconsistent among different brain regions. Studies of the Glx peak and its individual components were mostly targeted at GABA given the well-grounded observation of GABAergic dysfunction in depressed patients, but glutamate is also thought to have a prominent role in depression (Arnone et al. 2015).

The amygdala has a significant role in affective modulation, in particular for associating environmental stimuli with emotionally charged inputs including the processing of negative, unpleasant emotions (e.g., fear) and positive ones such as stimulus-reward processing (Baxter and Murray 2002).

Amygdala volume abnormalities do not appear consistently and may depend on the illness phase, medication use, and family history of major depressive disorder (MDD) (Kerestes et al. 2013; Schmaal et al. 2016). Preserved and increased volumes have been reported in those with recurrent episodes (Bellani et al. 2011), with the proposed explanation that increased brain-derived neurotrophic factor (BDNF) promotes neurogenesis and protects against glucocorticoid toxicity in the amygdala of medicated persons (Hamilton et al. 2008).

Details of amygdala-dependent plasticity in MDD remain to be characterized (Kuhn et al. 2014), but it is hypothesized that abnormalities in neuronal density, dendritic arborization, and synapse density may underlie impoverishment of glutamatergic neurotransmission, emphasizing that additional studies focusing on these relationships would be valuable (Yüksel and Öngür 2010).

The amygdala and dorsolateral prefrontal cortex (DLPFC) are tightly coupled (Clark et al. 2018; Connolly et al. 2017; Dannlowski et al. 2009; Murrough et al. 2011), and in depressed patients, the relationship between activities in these areas is decreased (Chen et al. 2008; Fales et al. 2009; Siegle et al. 2007; Tao et al. 2012).

Despite a growing knowledge base of amygdala involvement in affect processing, emotional reactivity, and chronic stress, this is one of the less studied brain regions in MDD (Marsden 2013). Amygdala MRS studies are particularly rare, due to technical complexities of small volume, high field inhomogeneity, and a shared boundary with the hippocampus (Nacewicz et al. 2012). Michael et al. longitudinally analyzed 1H-MRS parameters in the amygdala and adjacent anterior hippocampus in depressed patients before and after treatment and found a significant increase in NAA in patients who responded to electroconvulsive therapy (ECT) responders. A study performed by Kusumakar et al. (2001) analyzed Cho/Cr ratios in early onset depression and found significantly lower left amygdala values in patients compared to controls. Block et al. (2009) analyzed the amygdalar/hippocampal region (most of the hippocampal body and parts of its head, of the amygdala, and of the parahippocampal gyrus) in depressed patients and found individual AD treatment effect to correlate with an increase in NAA and Cho.

We previously reported no NAA changes in the left amygdala before and after an 8-week long AD treatment (Janović et al. 2014). Michael et al. (2003) analyzed MRS changes in the amygdala of treatment-resistant MDD patients and found a significant increase in NAA in ECT monotherapy responders, as well as after ECT was combined with AD therapy in non-responders. In all successfully treated patients, an increased Glx level was observed, whereas Cho and Cr remain unchanged. Ende et al. (2000), when applying ECT without AD, did not replicate NAA changes in the hippocampus after ECT, but described an increase in Cho in depressed patients compared to healthy controls (Ende et al. 2000). We were not able to identify any studies that compared 1H-MRS parameters in the amygdalar region after the acute treatment phase in relation to MDD episode recurrence.

The aim of this work was to assess the relationship between changes in 1H-MRS parameters at the start of the recovery phase and its duration (i.e., episode-free period) in patients suffering from recurrent depression receiving maintenance therapy. This approach is unique in correlating 1H-MRS changes at the beginning of the recovery period with clinical outcomes in the subsequent course of illness.

In our previous research on the same cohort, we showed that decreases in Cho/Cr and NAA/Cr ratios in the DLPFC after recovery were related to a higher risk of a recurrent episode during maintenance therapy; specifically, a decrease in Cho/Cr was associated with 2.7-fold higher risk (Henigsberg et al. 2017). Now, we hypothesize that our findings in the DLPFC study would be in contrast to those in regions oppositely involved in negative affective processing. Another, even self-limiting, factor in the selection of amygdalar region as the region of interest in the present study was that, among regions involved in affective processing, we only have raw data conformant to the same metabolites, study subjects, and periods for the amygdalar region.

Being aware of obstacles in interpreting 1H-MRS findings of the amygdalar area as region specific, we hypothesize that there is an increase in Cho levels, without significant NAA increase, in the amygdalar region during the recovery phase. Such changes would be concordant with the assumption of continued membrane consolidation without brain structural changes that would indicate enhanced arborization or other significant structural changes. This hypothesis is also in accordance with amygdala-PFC restructuring observed in volumetric and functional research.

We also assumed that the rise in Glx peak would be associated with a greater recurrence risk, as glutamate is the highest constituent of the Glx peak and amygdalar levels of this neurotransmitter decrease after depression treatment. We expect those changes to be observable if the hippocampus-originating signal does not obscure changes we expect to originate from the amygdala. Still, changes in Glx peaks were not included in our primary hypothesis, knowing the general difficulties in collecting and interpreting that metabolite in MRS (Nacewicz et al. 2012) and especially in the amygdala.

## Methods

This study identified participants among patients who had already participated in research of therapeutic response predictors. The patients had to fulfill the following inclusion criteria: diagnosis of unipolar depression (single or recurrent episode) diagnosed by a qualified psychiatrist based on International Classification of Diseases 10 (ICD-10) and confirmed by Mini international Neuropsychiatric Interview 5.0 (MINI v. 5.0.), two MRS acquisitions, no other psychiatric diagnoses, no significant somatic diagnoses requiring pharmacotherapy, on AD monotherapy during the study period with permitted stable dose of benzodiazepine treatment, not receiving any other therapy (e.g., psychotherapy, transcranial magnetic stimulation, etc.). ADs were prescribed in a stable dose, as reached over the remission period. All the patients participating in the study previously signed informed consent for long-term evaluation. The study was conducted in accordance with the Declaration of Helsinki and was approved by the Ethics Committees of the School of Medicine, University of Zagreb and Polyclinic Neuron.

The patients were followed up from depressive symptom remission and entrance into the recovery phase (recovery defined as remission of depressive symptoms for 4 consecutive months) until the recurrence of depression. Depressive symptom remission was confirmed by a Montgomery-Åsberg rating Scale (MARDS) score ≤ 10. The first MRS acquisition was scheduled at the beginning of the recovery phase and the second one 6 months later. All 48 patients completed both MRS scans. During the whole study period, patients had regular check-ups by their treating psychiatrist that included the psychiatric interview and MARDS evaluation. The check-ups were required at least every 6 months, but most patients had regular monthly doctor visits to obtain the necessary AD therapy via national health insurance.

**Fig. 1:**
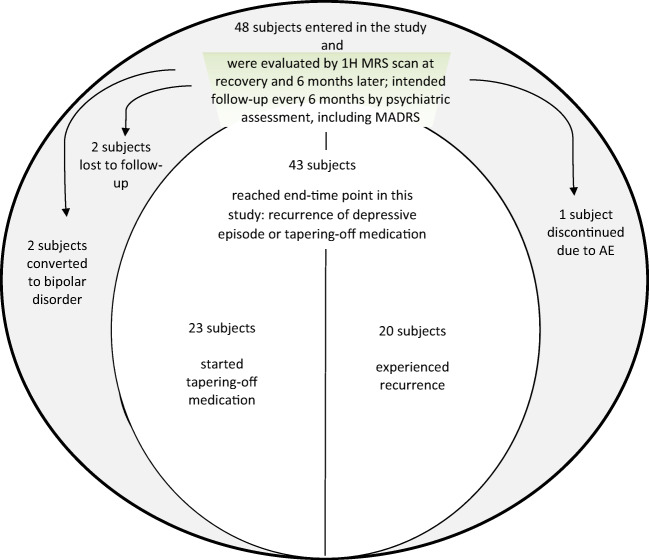
Study schematic

Five patients were excluded from the study due to conversion to bipolar disorder (2 patients), adverse events (1 patient suffered from serotonergic syndrome), or loss to follow-up (2 patients). Of the remaining 43 patients, one group had recurrence of depressive symptoms while on maintenance therapy, whereas the other group experienced a sufficiently long recovery to be tapered off the prescribed pharmacotherapy. For the purpose of this study, discontinuation was defined as the first time point in the study when the AD medication was tapered off (Fig. 1).

### 1H-MRS analyses

**Fig. 2:**
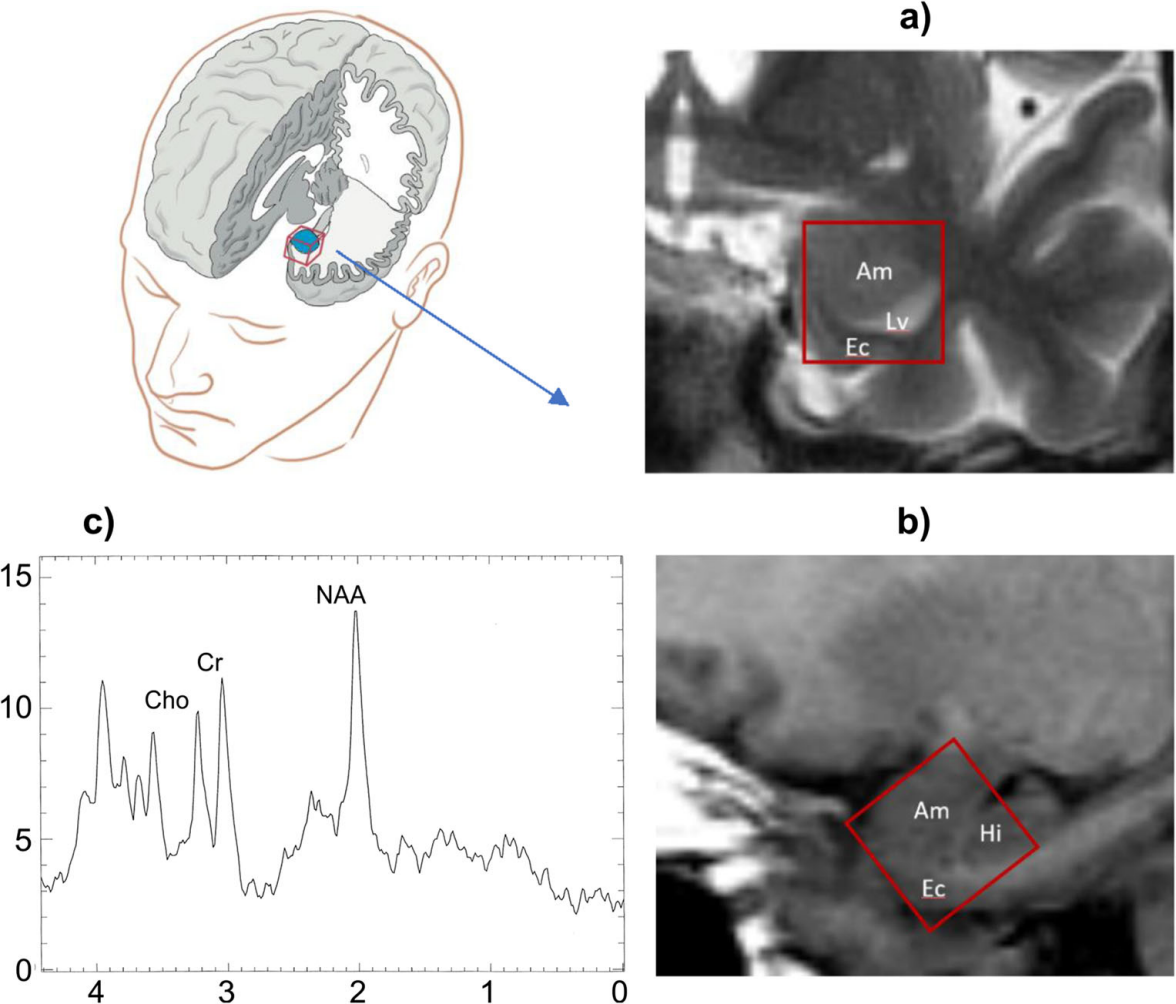
Voxel positioning, brain structures adjacent to amygdala in coronal plane (a), sagittal plane (b), and typical spectrum acquired from amygdala region (c). Am amygdala, Lv temporal horn of lateral ventricle, Ec entorhinal cortex, Hi head of hippocampus, Cho choline, Cr creatine, NAA N-acetyl aspartate (illustration by Milan Rados, neuroradiologist)

1H-MRS was performed after the psychiatric evaluation. All 1 H-MRS data acquisition in this study was performed on a 2.0 T system (Gyrex 2T-Prestige, GEMS/Elscint, Haifa, Israel) using a quadrature head coil. The subject was positioned supine on head foam pads to prevent head movements. The intersection of the frontal bone and two nasal bones (nasion) served as a landmark. Imaging was performed by a radiologist experienced in MRS acquisition. Standard T1 and T2 sequences completed in the coronal, sagittal, and axial planes and covering the entire brain were acquired for each subject to account for the effect of spectral localization and exclude possible structural brain damage. The spectroscopic volume of interest (VOI), 15 × 15 × 15 mm, was selected in the left amygdala region. The VOI was positioned to maximize amygdala content and avoid brain structures that could possibly interfere with magnetic field homogeneity (e.g., central cerebral arteries in front of the amygdala and medially positioned bone structures). The VOI contained the amygdala, as the primary target, but also part of the parahippocampal gyrus, hippocampus head, and minor parts of the temporal horn of the lateral ventricle. An example of voxel positioning, brain structures adjacent to target VOI and typical spectrum is shown in Fig. 2.

1H-MRS was performed using a point-resolved spectroscopy sequence (1500/35 [repetition time/echo time]), with 100 averages. Each spectrum was re-evaluated for peak NAA (at 2.02 ppm), Cho (at 3.2 ppm), Glx (at 2.2–2.4 ppm), and Cr (at 3.03 ppm). The absolute NAA, Cho, and Glx values and their ratios to Cr were used for the analyses.

Analyses of the spectral dataset were performed using the software package supplied by the manufacturer of the magnetic resonance system (Gyrex 2T-Prestige, GEMS/Elscint).

### Statistical analysis

The primary variable of interest in our research was time survived without an episode, specifically if differences in metabolite markers observed early in recovery would sustain as a risk/protective marker for a more distant time period. For this study, we employed the Cox proportional hazard (CPH), since it is suitable in situations when either the form of the true hazard function is unknown or is complex and assumes that predictors act multiplicatively on the hazard function but does not assume that the hazard function is of any particular form (Harrell 2001). This method accounts for both for survival time and censored information, while simultaneously assessing the effects of several risk/protective factors over time. Steps in the CPH model are described in detail in the Results section. We performed CPH analysis in two ways: first by using absolute changes of metabolites to Cr ratios as inputs, and second by dichotomization of those inputs (rise or fall between measurements).

Prior to displaying the CPH analysis results, to illustrate directions of changes in metabolite levels, and for the ease of comprehension of CPH results, we performed general linear model (GLM) multivariate analysis of metabolite levels in relation to the outcome of reaching the tapering period without a recurrence (sustained or recurred conditions). We performed GLM with two levels of within-subject factor (measurement time) and three levels of metabolite to Cr ratios. We considered multivariate testing appropriate as changes across levels (measurement times) were correlated across subjects.

Statistical analysis was performed in version 13 of the Statistica software package (TIBCO Software Inc., 2017). The level of statistical significance was set to 0.05.

## Results

In total, 48 subjects entered the study. Patients underwent two 1H-MRS scans of the left amygdala region: one at the beginning of recovery and another 6 months later. In the later period, they were followed up every 6 months until the endpoint, which was defined as a time either until the start of medication discontinuation (i.e., tapering-off medication in 23 subjects) or until recurrence of the next depressive episode (20 subjects). For the remaining five patients, data on type and time of cutoff point were missing. Among them, two patients were lost to follow-up, two converted to bipolar disorder, and one patient abruptly stopped medication due to an adverse event (serotonergic syndrome).

Thirty female and 18 male patients entered the study. The type and time of endpoint are missing for 4 female and 1 male patients, while the remaining 26 female and 17 male patients were followed until the tapering phase or episode onset. Demographic characteristics and current episode descriptors are displayed in Table 1.

**Table 1:** Demographics and current episode descriptors

	Sustained antidepressant response		Episode recurrence		
	*n* = 23		*n* = 20		
Variable	Mean	SD	Mean	SD	p
Age at first evaluation	44.48	13.23	43.58	11.59	0.8700
Age at disorder onset	28.21	7.75	27.59	8.98	0.8088
Years with depression	16.27	10.15	15.99	11.13	0.9328
No. of prior episodes	3.78	1.73	4	2.41	0.7332
Months to remission	5.93	1.4	5.81	1.65	0.7945
MADRS at episode onset	25.79	4.13	25.9	4.07	0.9305
MADRS at recovery onset phase	5.4	1.33	5.61	1.07	0.5696
MADRS 6 months after recovery onset	5.55	0.88	5.29	1.41	0.4575
MADRS at the visit prior to the last evaluation	5.34	1.1	5.44	1.27	0.7733
MADRS at the last evaluation	5.32	0.87	20.3	1.44	< 0.0001

**Fig. 3:**
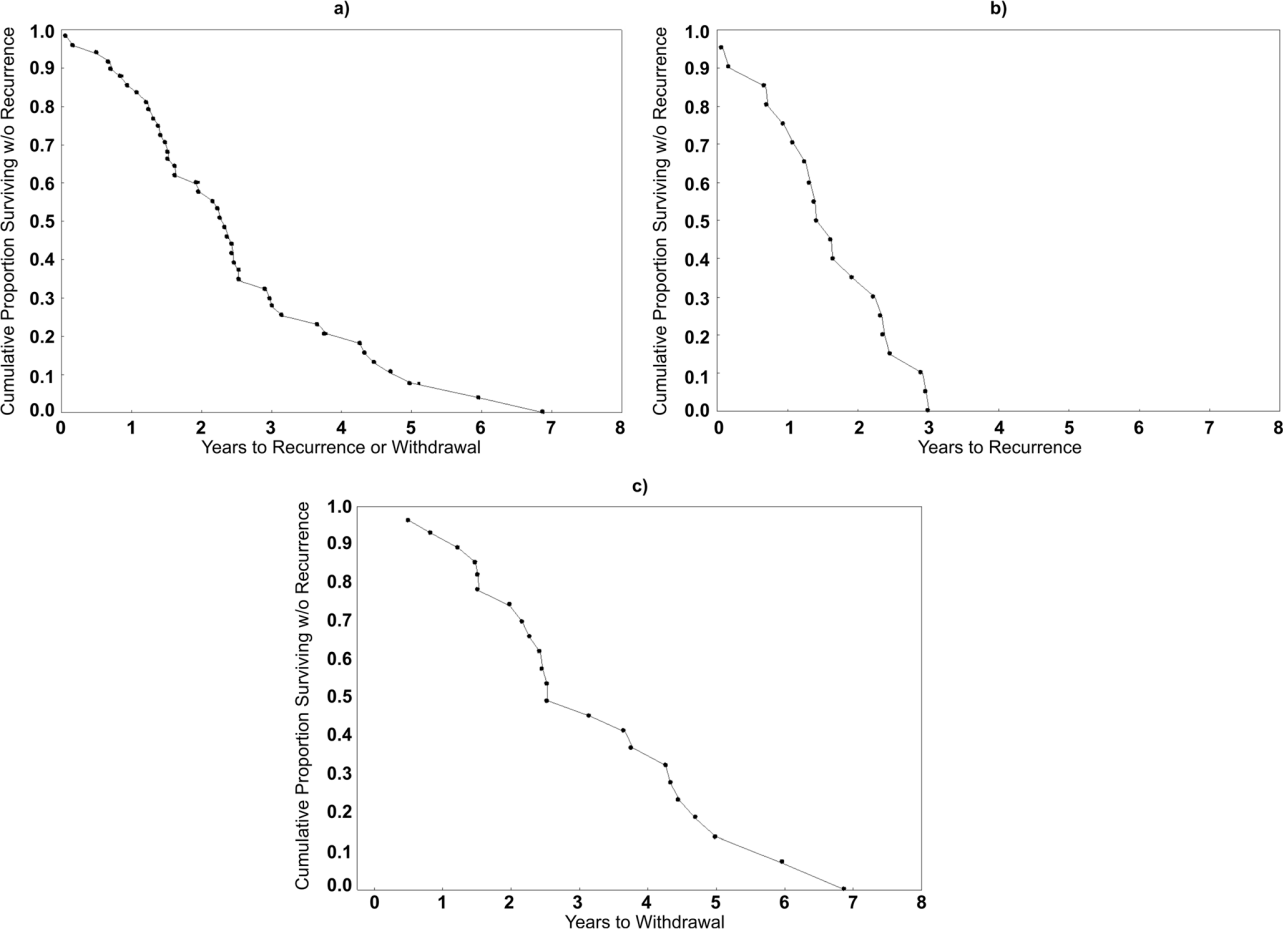
Kaplan–Meier plots of time to recurrence or start of tapering-off medication for all subjects in the study (**a**), time to recurrence for patients who relapsed during maintenance therapy (**b**), and time to withdrawal of medication for those who sustained without relapse over the course of maintenance therapy (**c**)

Patients went without a recurrent episode on average for 2.42 years (SD = 1.53), and this was expectedly higher in the group that sustained until the tapering period (3.03; SD = 1.67) compared to patients who relapsed over time on full-dose medication (1.63; SD = 0.89). Kaplan–Meier curves of survival without another episode are displayed in Fig. 3.

**Fig. 4:**
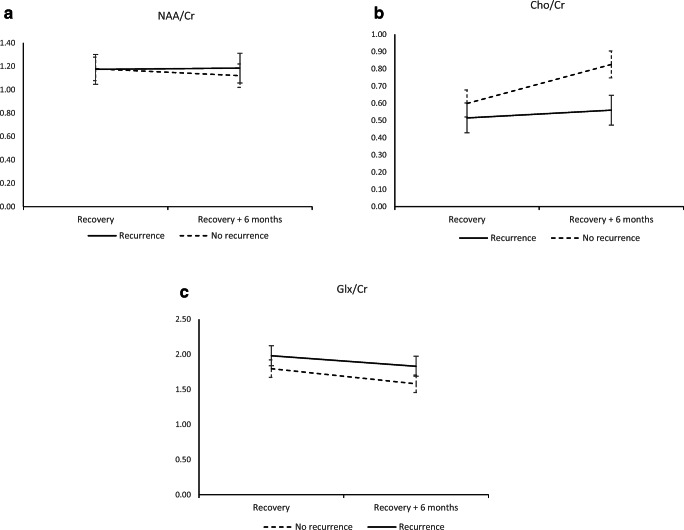
MRS measurements of **a** NAA/Cr, **b** Cho/Cr, and **c** Glx/Cr. Recurrence—followed up until the start of AD medication discontinuation. No recurrence—followed-up until recurrence of a depressive episode. Horizontal axis: recovery—start of the recovery phase; recovery + 6 months—start of the recovery phase + 6 months. Error bars denote 95% CIs of means

GLM repeated measures analysis was conducted to compare the effect of metabolite levels on recurrence of a depressive episode before reaching tapering-off medication. There was a significant effect of a three-way interaction of measurement time, metabolite, and recurrence in a multivariate test, Wilks’ lambda = 0.857, *F*(2,40) = 3.348, *p* = 0.045. The directions of changes in metabolite levels based on the status of recurrence during maintenance therapy are shown in Fig. 4.

In preparation for CPH analysis, we confirmed the absence of the time-varying effect, to ensure that observed metabolite changes were not correlates of neurochemical changes already occurring in a not yet clinically manifested episode. Proportional hazard assumptions were verified by constructing a product between the variable and linear function of time, adding an interaction term, and testing for its significance. We also assessed the proportionality assumption by visually checking plots of the log–log [S(t)] functions and confirmed constant hazard ratios.

In the initial model, we added variables related to our neurometabolites (values at the first scan and differences in the second scan), and three disorder and three current episode descriptors confirmed from literature sources as indicators of the severity and longitudinal course of the current episode.

The initial CPH model had nominal statistical significance (− 2 log likelihood = 101.802; *χ*^2^ = 33.147; *df* = 12; *p* = 0.0009), but evaluation of beta coefficients, risk ratios with their 95% confidence intervals (CIs), *t* value approximations, and Wald statistics indicated significant contributions of five variables, all of them related to our neurometabolites of interest (Table 2). Among these, three measure metabolite levels at recovery, reflecting the significant differences already observed in these levels at the first scan. Changes in Cho and Glx from the first to the second scans were also statistically significant.

**Table 2:** Parameter estimates of the initial model

Factor	Relative risk (95% CI)	*p*
1H-MRS metabolite levels at recovery		
NAA/Cr	0.0314 (0.0011–0.9367)	0.0458
Cho/Cr	0.0054 (0.0002–0.1951)	0.0043
GLX/Cr	68.9960 (2.4546–1939.3790)	0.0129
1H-MRS metabolite changes 6 months after recovery		
NAA/Cr	1.1188 (0.1714–7.3025)	0.9066
Cho/Cr	0.0007 (0–0.0263)	0.0001
GLX/Cr	18.0679 (1.2155–268.5807)	0.0356
Current episode descriptors		
Months to remission	1.4762 (0.9179–2.3741)	0.1081
MADRS improvement at recovery	1.0284 (0.8816–1.1996)	0.7216
MADRS change 6 months after recovery	0.8131 (0.5219–1.2668)	0.3604
Disorder course descriptors		
Age at onset	0.9961 (0.9151–1.0844)	0.9289
Years with depression	1.0144 (0.9487–1.0846)	0.6754
No. of prior episodes	1.1749 (0.8297–1.6637)	0.3638

To further evaluate individual variable contributions, we entered five identified variables into the CPH forward stepwise likelihood ratio model, which consisted of two steps.

In the first step, Cho change at 6 months after recovery was included [*B* = − 2.139; SE = 1.006; Wald = 4.523; *df* = 1; *p* = 0.033; Exp(*B*) = 0.118]. The final model consisted of two variables: Cho level at recovery [*B* = − 4.735; SE = 1.405; Wald = 11.349; *df* = 1; *p* = 0.001; Exp(*B*) = 0.009] and Cho change 6 months after recovery [*B* = − 4.550; SE = 1.314; Wald = 11.988; *df* = 1; *p* = 0.001; Exp(*B*) = 0.011], so both variables included related to Cho levels (− 2 log likelihood = 118.867; *χ*^2^ = 16.032; *df* = 2; *p* = 0.0032). Parameter estimates of the final model are displayed in Table 3.

**Table 3:** Parameter estimates of the final model

Factor	Relative risk (95% CI)	*p*
Cho/Cr at recovery	0.0088 (0.0006–0.1381)	0.0008
Cho/Cr change 6 months after recovery	0.0106 (0.0008–0.1389)	0.0005
After dichotomization (only rise or fall monitored)		
Cho/Cr changes 6 months after recovery		
Cho/Cr decreases	1.0000	
Cho/Cr remains equal or rises	0.3156 (0.1301–0.7656)	0.0108

It is difficult to generalize a multivariable model based on ratio to Cr units. For this reason, and to present the results in a simplified way, we dichotomized the variable measuring change in Cho after 6 months. Even this univariate model with one dichotomized variable alone sufficed to produce significance (− 2 log likelihood = − 134.95; *χ*^2^ = 6.42; *df* = 1; *p* = 0.011). Subjects with a rise in Cho after recovery have a risk of subsequent episode, at any particular time point, that is only a third (31.56%) of that compared to subjects experiencing Cho decrease (HR = 0.32; 95% CI 0.13–0.77; *t* = − 2.55; *p* = 0.011).

MADRS score significantly improved from the beginning of an episode to the start of the recovery phase (20.3, SD = 4.1 in the group with recurrence vs. 20.4, SD = 3.8, *p* = 0.93, in the group that started therapy discontinuation). At the 6-month follow-up, MADRS changes were only minor: (0.2, SD = 1.7) on average in subjects who entered the therapy discontinuation period and (− 0.3, SD = 1.6) in those who experienced a recurrent episode.

Concerning ADs in treatment, escitalopram was the most frequently used medication—in 5 patients who entered the tapering-off period and in 6 patients who experienced a recurrent episode. In patients who experienced recurrence, other used ADs were sertraline (5), mirtazapine (2), fluoxetine (1), venlafaxine (1), reboxetine (1), citalopram (1), fluvoxamine (1), paroxetine (1), and imipramine (1). The tapering-off group used fluoxetine (4), sertraline (4), venlafaxine (3), amitriptyline (2), reboxetine (1), citalopram (1), fluvoxamine (1), paroxetine (1), and mirtazapine (1).

Five of 23 (21.7%) patients who sustained until AD discontinuation and 4 of 20 (20%) who relapsed in continuation phase were under a stable dose of medication in the benzodiazepine class. In the sustained group, no apparent differences were observed in changes of NAA levels (− 0.0811, SD = 0.5697 in the benzodiazepine group vs. − 0.0519, SD = 0.3458 in others), Cho levels (0.2134, SD = 0.1409 in the benzodiazepine group vs. 0.2302, SD = 0.2965 in others), or Glx levels (− 0.1541, SD = 0.1669 in the benzodiazepine group vs. − 0.233, SD = 0.5029 in others). These differences were not observed in relapsed patient NAA levels (0.008, SD = 0.3473 in the benzodiazepine group vs. 0.0118, SD = 0.3997 in others), Cho levels (0.061, SD = 0.2495 in the benzodiazepine group vs. 0.041; SD = 0.2171 in others), or Glx levels (− 0.06, SD = 0.4964 in benzodiazepine group vs. − 0.1724, SD = 0.4681 in others). None of the 5 patients lost to follow-up received a stable dose of medication from the benzodiazepine class.

## Discussion

To our knowledge, this is the first study to identify 1H-MRS correlates of recurrence risk in the amygdalar region in patients with depression on maintenance therapy during the recovery phase. We demonstrated that Cho level in the left amygdala at recovery onset and changes 6 months later in recurrent MDD patients receiving AD continuation therapy predict a longer episode-free period, and the effect is not time varying (i.e., decreased Cho could not be attributed to a chemical change in already started but not yet clinically manifested episode). We also observed a significant Glx decrease in non-recurrent patients.

Even when only analyzing the rise or fall of Cho in the recovery phase, regardless of the magnitude, a rise in Cho is associated with only one third the risk (HR = 0.32; 95% CI 0.13–0.77) at any time compared to the risk in patients experiencing no change or fall of Cho.

A direct comparison of our results with previous studies that measured metabolite levels in the amygdalar region is not possible because of considerable differences in the populations studied, employed treatments, periods between MRS scans, scanning techniques, and differences in voxel position.

The rise of Cho-containing compounds in the amygdalar region was notably higher in patients with better prognoses. Meanwhile, NAA did not change considerably. As NAA is considered a neuron-specific marker of integrity and neuronal energetic processes, and Cho is a marker of membrane metabolism and turnover, our findings are in line with previous research showing variations in neuronal plasticity after AD therapy.

Differences in Glx levels already existed at the first scan and increased further 6 months later. The decrease in Glx was considerably higher in the group with good prognosis. This trapezoid-like pattern indicates the possibility that changes we observed in recovery existed even before, and we simply observed the continuation of this trend. This does not necessarily imply that changes of the same metabolites started from the beginning with the same magnitude. We reiterate that the period of our research was *after* acute and remission phases when patients still remained on the same AD therapy used earlier in the treatment.

We were only able to measure composite Glx peaks, not separate GABA peaks, so we could not unequivocally relate observed Glx changes to any particular metabolite. Analyzed voxels inevitably contained some portion of brain tissue other than the amygdala, so we cannot claim that the Cho turnover and Glx decrease were amygdala specific.

However, we made our best effort to position VOIs to contain the largest volumes of amygdala nuclei. Considering all the limitations associated with collecting and analyzing amygdalar region data (Brierley et al. 2002), we could claim that Cho and Glx compositions changed considerably between the two periods, irrespective of brain tissue composition in the voxels. Still, analyzed voxels inevitably contained some structures adjacent to the amygdala. The structures most likely to be captured together with the amygdala are illustrated in Fig. 2.

Lack of a water reference dataset is a limitation of our study, and normalization to Cr was the only feasible option. Still, there was noticeable interscan variability of the Cr peak, with a coefficient of variability of 11.3%.

It seems reasonable to assume that Glx change is largely attributable to glutamate, as it is the most prominent component of Glx peak and the most abundant brain neurotransmitter. If hippocampal tissue accounted for a considerable portion of the analyzed voxel, one could contemplate that Glx decrease would reflect correction of the glutamatergic hyperactivity present in depression. However, the observed Glx decrease is more congruent with the hypothesis and findings of decreased glutamatergic activity in the amygdala during recovery from depression, but it is highly speculative to relate our finding to any specific brain structure, as glutamate/glutamine even in postmortal analyses of the same brain regions in depression are not uniform and differ by symptom severity (Zhao et al. 2016).

Several confounding facts complicate the interpretation of eventual changes in glutamate levels. Glutamatergic downregulation is not necessarily linked to changes in neuronal function, since glutamate is also present in astrocytes (Márquez et al. 2009). It cannot be excluded that glutamate is only mirroring changes in neurotransmission of mineralocorticoid and glucocorticoid receptors. It is also possible that changes in glutamatergic neurotransmission are at least partially treatment-specific, as ADs used for a longer term cause considerable increases in AMPA receptor subunit protein expression (Barbon et al. 2011). These changes occur regardless of serotonin or noradrenaline AD selectivity, but it is yet unknown how long these changes last. Hypothetically, upregulation of receptors may be related to changed ligand levels.

A concomitant Cho increase and Glx decrease, in absence of NAA changes, may indicate a shift towards “non-glutamate”-mediated neuronal function, which would support the glutamate/GABA hypothesis of depression. Decreasing from high to lower levels of the main excitatory neurotransmitter may be linked to decreased negative emotive processing, which occurs in all regions involved in affective processing, except for certain PFC regions, and tend to normalize with treatment (Jaworska et al. 2015). However, evidence for the neurorestorative effects of AD treatment in humans is still rare, and it is difficult to directly relate an eventual neurorestorative effect to a particular chemical substance or class used in therapy.

We expected to observe a change in Cho; however, that hypothesis was based on consistent findings of increased Cho prior to the recovery phase. We assume that the observed Cho change is consequent to a PC-to-GPC-mediated synthesis-to-breakdown overbalance, reflecting consolidation rather than increase in neuronal mass, volume, or activity. This would be congruent with the amygdala-to-DLPFC functional connectivity shift reported in neuroimaging studies.

When changes in metabolite levels in the amygdalar region are contrasted to our previously reported DLPFC findings, our results provide further support of an amygdala-DLPFC interconnectivity shift in MDD on the biochemical level. Whereas our results indicate excitatory diminishment in the amygdala and restructuring without an increase in neuronal density or metabolism, we had earlier shown opposing directions of change, presumably consequent to regeneration processes in the DLPFC.

There are several limitations of our study. Like all MRS studies using the technique applied in our study, it is not feasible to separate neuronal and glial signals. Therefore, all our findings might not be attributable solely to neuronal changes. Astrocytes and oligodendrocytes are directly or indirectly involved in the life-cycles of metabolites analyzed in this research. So, the terms used here to describe changes, like *integrity*, *viability*, *plasticity*, or *restructuring* refer to all cellular entities composing an analyzed voxel.

In discussing our results, we presume that analyzed metabolites are directly related to pathophysiological processes in determining the course of depression. On the other hand, it is also plausible they are only indirect correlates of yet unknown processes.

As mentioned earlier, difficulty delineating the amygdala is a considerable limitation of this study. This is not specific to our research but relates to all types of MRI measurements claiming to show amygdala volumes or structures. There is high within-subject variability in measuring metabolites in the amygdala, and Glx in particular, since less abundant metabolites are difficult to quantify due to coupling and peak overlap (Nacewicz et al. 2012). However, we believe it is unlikely that the high differences in Glx we observed in this study were false positives, as within-group variances are in the range of a more advanced MR technique we are currently using. Real within-subject differences and those not caused by errors in measurement could be high in the developing brain but could be considered negligible in our sample, as enlargement of any particular structure between two scans is unlikely in adult subjects.

Another limitation is related to study design and endpoint definition. By setting the endpoint to the start of tapering-off medication and episode recurrence, we superimposed future knowledge to a historical time point. This was inevitable in our present research, in order to maintain the same study conditions in both groups. For an eventual definition of clinical outcome predictors, a study would also have to consider the outcomes in terms of recurrence after medication discontinuation. However, a considerably larger sample would be required to accurately evaluate covariates of that type.

There was a limited range of episode severity in our sample, and we could not perform an analysis of the relationship of baseline severity of an episode to 1H-MRS metabolite changes. We did not include several identified risk factors of recurrence such as the presence of residual depressive symptoms prior to the current episode, previous history of the dysthymic disorder, and family history of recurrent depression. We were aware of this limitation prior to the study, but we considered that the information was not fully reliable when sourced from a patient rather than medical documentation.

Although the sample size was relatively large for the research of this type, it was underpowered to detect meaningful clinical sensitivity and specificity. Replication of our findings is certainly necessary, preferably in a prospective stratified design, with a wider battery of psychometric instruments and more frequent MRS evaluations, ranging from the beginning of the episode until its resolution. More frequent data collection would eliminate potential temporal fluctuations and improve model specificity and sensitivity.

More advanced MR techniques with refined post-processing will reduce the margins of error in defining candidate MRS predictors of MDD recurrence risk. Reproduction of our study design in a larger sample and with more frequent metabolite measurements is needed to validate the use of early 1H-MRS metabolite changes as biomarkers of the future longitudinal course of depression.

## References

[CR1] American Psychiatric Association (APA) (2010). Practice guideline for the treatment of patients with major depressive disorder. 3rd edn..

[CR2] Arnone D, Mumuni AN, Jauhar S, Condon B, Cavanagh J (2015). Indirect evidence of selective glial involvement in glutamate-based mechanisms of mood regulation in depression: meta-analysis of absolute prefrontal neuro-metabolic concentrations. Eur Neuropsychopharmacol.

[CR3] Barbon A, Caracciolo L, Orlandi C, Musazzi L, Mallei A, La Via L, Bonini D, Mora C, Tardito D, Gennarelli M, Racagni G, Popoli M, Barlati S (2011). Chronic antidepressant treatments induce a time-dependent up-regulation of AMPA receptor subunit protein levels. Neurochem Int.

[CR4] Baxter MG, Murray EA (2002). The amygdala and reward. Nat Rev Neurosci.

[CR5] Bellani M, Baiano M, Brambilla P (2011). Brain anatomy of major depression II. Focus on amygdala. Epidemiol Psychiatr Sci.

[CR6] Bernier D, Barthxa R, Devarajan S, Macmaster FP, Schmidt MH, Rusak B (2009). Effects of overnight sleep restriction on brain chemistry and mood in women with unipolar depression and healthy controls. J Psychiatry Neurosci.

[CR7] Block W, Träber F, von Widdern O, Metten M, Schild H, Maier W, Zobel A, Jessen F (2009). Proton MR spectroscopy of the hippocampus at 3 T in patients with unipolar major depressive disorder: correlates and predictors of treatment response. Int J Neuropsychopharmacol.

[CR8] Brierley B, Shaw P, David AS (2002). The human amygdala: a systematic review and meta-analysis of volumetric magnetic resonance imaging. Brain Res Brain Res Rev.

[CR9] Caverzasi E, Pichiecchio A, Poloni GU, Calligaro A, Pasin M, Palesi F, Castellazzi G, Pasquini M, Biondi M, Barale F, Bastianello S (2012). Magnetic resonance spectroscopy in the evaluation of treatment efficacy in unipolar major depressive disorder: a review of the literature. Funct Neurol.

[CR10] Chen CH, Suckling J, Ooi C, Fu CHY, Williams SCR, Walsh ND, Mitterschiffthaler MT, Pich EM, Bullmore E (2008). Functional coupling of the amygdala in depressed patients treated with antidepressant medication. Neuropsychopharmacology..

[CR11] Clark DL, Konduru N, Kemp A, Bray S, Brown EC, Goodyear B, Ramasubbu R (2018). The impact of age of onset on amygdala intrinsic connectivity in major depression. Neuropsychiatr Dis Treat.

[CR12] Connolly CG, Ho TC, Blom EH, LeWinn KZ, Sacchet MD, Tymofiyeva O, Simmons AN, Yang TT (2017). Resting-state functional connectivity of the amygdala and longitudinal changes in depression severity in adolescent depression. J Affect Disord.

[CR13] Dannlowski U, Ohrmann P, Konrad C, Domschke K, Bauer J, Kugel H, Hohoff C, Schöning S, Kersting A, Baune BT, Mortensen LS, Arolt V, Zwitserlood P, Deckert J, Heindel W, Suslow T (2009). Reduced amygdala-prefrontal coupling in major depression: association with MAOA genotype and illness severity. Int J Neuropsychopharmacol.

[CR14] Ende G, Braus DF, Walter S, Weber-Fahr W, Henn FA (2000). The hippocampus in patients treated with electroconvulsive therapy: a proton magnetic resonance spectroscopic imaging study. Arch Gen Psychiatry.

[CR15] Fales CL, Barch DM, Rundle MM, Mintun MA, Mathews J, Snyder AZ, Sheline YI (2009). Antidepressant treatment normalizes hypoactivity in dorsolateral prefrontal cortex during emotional interference processing in major depression. J Affect Disord.

[CR16] Frahm J, Bruhn H, Gyngell ML, Merboldt KD, Hänicke W, Sauter R (1989). Localized high-resolution proton NMR spectroscopy using stimulated echoes: initial applications to human brain in vivo. Magn Reson Med.

[CR17] Hamilton JP, Siemer M, Gotlib IH (2008). Amygdala volume in major depressive disorder: a meta-analysis of magnetic resonance imaging studies. Mol Psychiatry.

[CR18] Harrell FE (2001). Cox proportional hazards regression model. Regression modeling strategies. Springer series in statistics.

[CR19] Henigsberg N, Bajs M, Hrabac P, Kalember P, Rados M, Rados M (2011). Changes in brain metabolites measured with magnetic resonance spectroscopy in antidepressant responders with comorbid major depression and posttraumatic stress disorder. Coll Antropol.

[CR20] Henigsberg N, Šarac H, Radoš M, Radoš M, Ozretić D, Foro T, Erdeljić Turk V, Hrabač P, Bajs Janović M, Rak B, Kalember P (2017). Lower choline-containing metabolites/creatine (Cr) rise and failure to sustain NAA/Cr levels in the dorsolateral prefrontal cortex are associated with depressive episode recurrence under maintenance therapy: a proton magnetic resonance spectroscopy retrospective cohort study. Front Psychiatry.

[CR21] Janović M, Kalember P, Janović Š, Hrabač P, Grošić P, Grošić V, Radoš M, Henigsberg N (2014). No change in N-acetyl aspartate in first episode of moderate depression after antidepressant treatment: 1H magnetic spectroscopy study of left amygdala and left dorsolateral prefrontal cortex. Neuropsychiatr Dis Treat.

[CR22] Jaworska N, Yang XR, Knott V, MacQueen G (2015). A review of fMRI studies during visual emotive processing in major depressive disorder. World J Biol Psychiatry.

[CR23] Kerestes R, Davey CG, Stephanou K, Whittle S, Harrison BJ (2013). Functional brain imaging studies of youth depression: a systematic review. Neuroimage Clin.

[CR24] Kuhn M, Höger N, Feige B, Blechert J, Normann C, Nissen C (2014). Fear extinction as a model for synaptic plasticity in major depressive disorder. PLoS One.

[CR25] Kusumakar V, MacMaster FP, Gates L, Sparkes SJ, Khan SC (2001). Left medial temporal cytosolic choline in early onset depression. Can J Psychiatr.

[CR26] Luborzewski A, Schubert F, Seifert F, Danker-Hopfe H, Brakemeier EL, Schlattmann P, Anghelescu I, Colla M, Bajbouj M (2007). Metabolic alterations in the dorsolateral prefrontal cortex after treatment with high-frequency repetitive transcranial magnetic stimulation in patients with unipolar major depression. J Psychiatr Res.

[CR27] Márquez J, Tosina M, de la Rosa V, Segura JA, Alonso FJ, Matés JM, Campos-Sandoval JA (2009). New insights into brain glutaminases: beyond their role on glutamatergic transmission. Neurochem Int.

[CR28] Marsden WN (2013). Synaptic plasticity in depression: molecular, cellular and functional correlates. Prog Neuro-Psychopharmacol Biol Psychiatry.

[CR29] Michael N, Erfurth A, Ohrmann P, Arolt V, Heindel W, Pfleiderer B (2003). Neurotrophic effects of electroconvulsive therapy: a proton magnetic resonance study of the left amygdalar region in patients with treatment-resistant depression. Neuropsychopharmacology.

[CR30] Moffett JR, Ross B, Arun P, Madhavarao CN, Namboodiri AM (2007). N-Acetylaspartate in the CNS: from neurodiagnostics to neurobiology. Prog Neurobiol.

[CR31] Murrough JW, Iacoviello B, Neumeister A, Charney DS, Iosifescu DV (2011). Cognitive dysfunction in depression: neurocircuitry and new therapeutic strategies. Neurobiol Learn Mem.

[CR32] Nacewicz BM, Angelos L, Dalton KM, Fischer R, Anderle MJ, Alexander AL, Davidson RJ (2012). Reliable non-invasive measurement of human neurochemistry using proton spectroscopy with an anatomically defined amygdala-specific voxel. Neuroimage.

[CR33] Petroff OA (1988). Biological 1H NMR spectroscopy. Comp Biochem Physiol B.

[CR34] Rush AJ, Kraemer HC, Sackeim HA, Fava M, Trivedi MH, Frank E, Ninan PT, Thase ME, Gelenberg AJ, Kupfer DJ, Regier DA, Rosenbaum JF, Ray O, Schatzberg AF, ACNP Task Force (2006). Report by the ACNP Task Force on response and remission in major depressive disorder. Neuropsychopharmacology.

[CR35] Sager TN, Topp S, Torup L, Hanson LG, Egestad B, Møller A (2001). Evaluation of CA1 damage using single-voxel 1H-MRS and un-biased stereology: can non-invasive measures of N-acetyl-asparate following global ischemia be used as a reliable measure of neuronal damage?. Brain Res.

[CR36] Schmaal L, Veltman DJ, van Erp TG, Sämann PG, Frodl T, Jahanshad N, Loehrer E, Tiemeier H, Hofman A, Niessen WJ, Vernooij MW, Ikram MA, Wittfeld K, Grabe HJ, Block A, Hegenscheid K, Völzke H, Hoehn D, Czisch M, Lagopoulos J, Hatton SN, Hickie IB, Goya-Maldonado R, Krämer B, Gruber O, Couvy-Duchesne B, Rentería ME, Strike LT, Mills NT, de Zubicaray GI, McMahon KL, Medland SE, Martin NG, Gillespie NA, Wright MJ, Hall GB, MacQueen GM, Frey EM, Carballedo A, van Velzen LS, van Tol MJ, van der Wee NJ, Veer IM, Walter H, Schnell K, Schramm E, Normann C, Schoepf D, Konrad C, Zurowski B, Nickson T, McIntosh AM, Papmeyer M, Whalley HC, Sussmann JE, Godlewska BR, Cowen PJ, Fischer FH, Rose M, Penninx BW, Thompson PM, Hibar DP (2016). Subcortical brain alterations in major depressive disorder: findings from the ENIGMA Major Depressive Disorder working group. Mol Psychiatry.

[CR37] Siegle GJ, Thompson W, Carter CS, Steinhauer SR, Thase ME (2007). Increased amygdala and decreased dorsolateral prefrontal BOLD responses in unipolar depression: related and independent features. Biol Psychiatry.

[CR38] Solomon DA, Keller MB, Leon AC, Mueller TI, Lavori PW, Shea MT, Coryell W, Warshaw M, Turvey C, Maser JD, Endicott J (2000). Multiple recurrences of major depressive disorder. Am J Psychiatry.

[CR39] Stanley JA (2002). In vivo magnetic resonance spectroscopy and its application to neuropsychiatric disorders. Can J Psychiatr.

[CR40] Stanley JA, Pettegrew JW, Keshavan MS (2000). Magnetic resonance spectroscopy in schizophrenia: methodological issues and findings — part I. Biol Psychiatry.

[CR41] Tao R, Calley CS, Hart J, Mayes TL, Nakonezny PA, Lu H, Kennard BD, Tamminga CA, Emslie GJ (2012). Brain activity in adolescent major depressive disorder before and after fluoxetine treatment. Am J Psychiatry.

[CR42] TIBCO Software Inc. (2018). Statistica (data analysis software system), version 13. http://tibco.com. Accessed 2 May 2019

[CR43] Tsai G, Coyle JT (1995) N-acetylaspartate in neuropsychiatric disorders. Prog Neurobiol 46:531–540 10.1016/0301-0082(95)00014-m 8532851

[CR44] van der Graaf M (2010). In vivo magnetic resonance spectroscopy: basic methodology and clinical applications. Eur Biophys J.

[CR45] Yüksel C, Öngür D (2010). Magnetic resonance spectroscopy studies of glutamate-related abnormalities in mood disorders. Biol Psychiatry.

[CR46] Zhang Y, Han Y, Wang Y, Zhang Y, Li L, Jin E, Deng L, Watts B, Golden T, Wu N (2015). A MRS study of metabolic alterations in the frontal white matter of major depressive disorder patients with the treatment of SSRIs. BMC Psychiatry.

[CR47] Zhao J, Verwer RW, van Wamelen DJ, Qi XR, Gao SF, Lucassen PJ, Swaab DF (2016). Prefrontal changes in the glutamate-glutamine cycle and neuronal/glial glutamate transporters in depression with and without suicide. J Psychiatr Res.

